# Supplementary material for: A GCN1-independent activator of the kinase GCN2
Source: eLife. 2026 Oct 27;14:RP108590. doi: 10.7554/eLife.108590 (PMC13713030; doi:10.7554/eLife.108590)
Supplement: Supplementary file 2 [file elife-108590-fig5-data1.pdf]

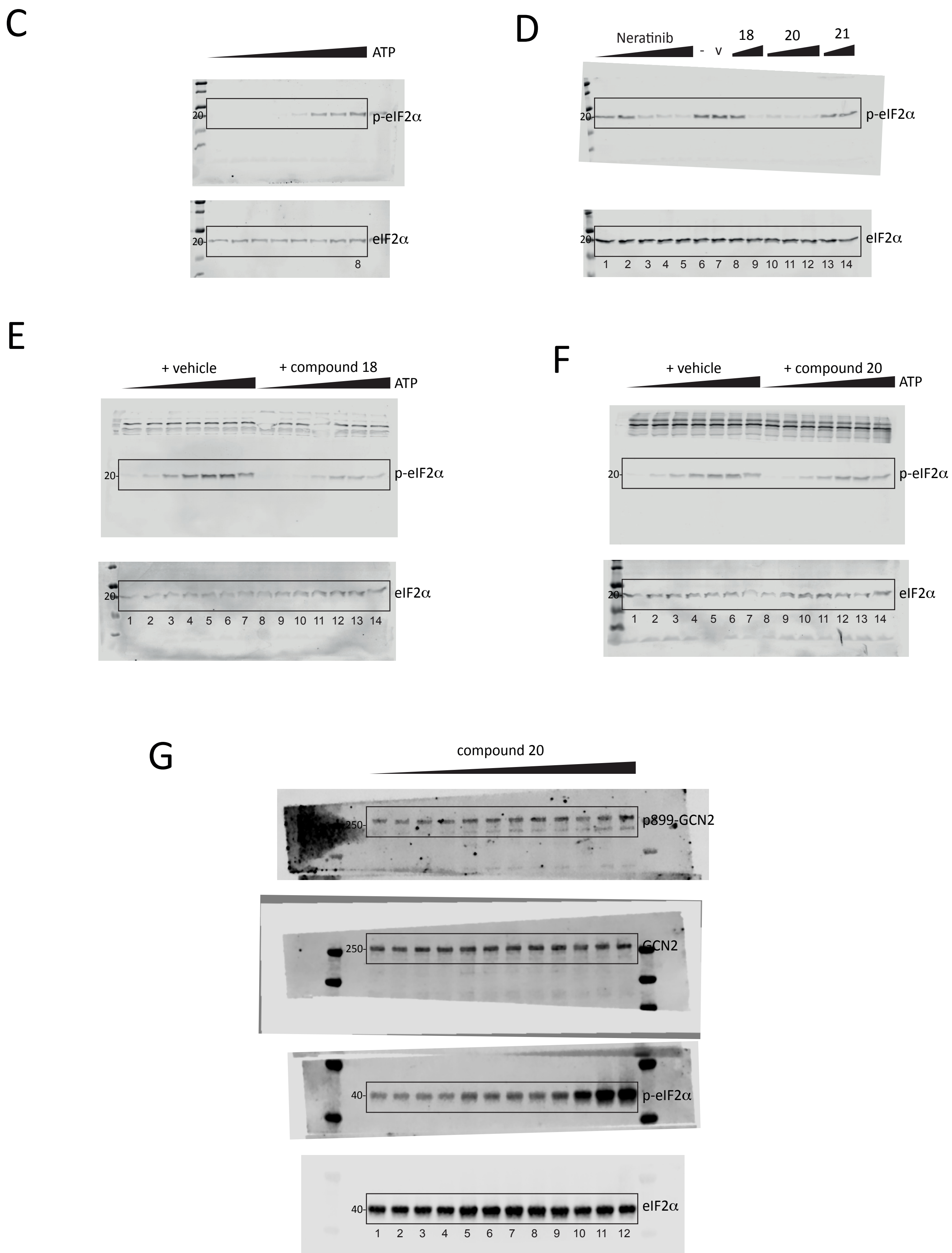

Figure 5, Source Data 1. Original membranes for panels C-G. Biorad prestained markers were used.
