# Supplementary material for: A GCN1-independent activator of the kinase GCN2
Source: eLife. 2026 Oct 27;14:RP108590. doi: 10.7554/eLife.108590 (PMC13713030; doi:10.7554/eLife.108590)
Supplement: Peer review history

# Editor report: eLife Assessment

**DOI:** 10.7554/eLife.108590.3.sa0

**Authors:** Julien Roche

**Affiliations:** Iowa State University United States

**Keywords:** Compelling, Important

This **important** study presents a cell-based screen for small-molecule activators of GCN2, an eIF2α kinase that regulates the Integrated Stress Response under diverse stress conditions. The authors identify a compound as a potent GCN2 activator with GCN1-independent activity under the tested conditions, providing a new pharmacological tool for probing GCN2 regulation. The revised manuscript provides **compelling** support for the central conclusions through a clearer description of the screening workflow, extended kinetic analyses, and demonstration that the identified compound causes a GCN2-dependent reduction in protein synthesis.

# Referee report: Reviewer #1 (Public review)

**DOI:** 10.7554/eLife.108590.3.sa1

Summary:

This manuscript describes a chemical screen for activators of the eIF2 kinase GCN2 (EIF2AK4) in the integrated stress response (ISR). Recently, reported inhibitors of GCN2 and other protein kinases have been shown at certain concentrations to paradoxically activate GCN2. The study uses CHO cells and ISR reporter screens to identify a number of GCN2 activator compounds, including a potent "compound 20." These activators have implications for the development of new therapies for ISR-related diseases. For example, although not directly pursued in this study, these GCN2 activators could be helpful for the treatment of PVOD, which is reported for patients with certain GCN2 loss-of-function mutations. The identified activators are also suggested to engage with the GCN2 directly and can function devoid of GCN1, a co-activator of GCN2.

Strengths:

The manuscript appears to be a largely rigorous study that flows in a logical manner. The topic is interesting and significant.

Weaknesses:

Portions of the manuscript are not fully clear. There are some experimental presentation and design concerns that should be addressed to support the stated conclusions.

# Referee report: Reviewer #2 (Public review)

**DOI:** 10.7554/eLife.108590.3.sa2

Summary:

In this manuscript Zhu, Emanuelli and colleagues describe a novel pharmacological activator of the Integrated Stress Response kinase GCN2. The work is conclusive and biochemically solid. This work significantly adds to the pharmacological arsenal targeting the ISR and in particular GCN2.

Strengths:

Strong biochemistry, novel molecular activator of GCN2 (GCN1 independent).

Weaknesses:

Rationale for the screen not exploited in the results (e.g. pathogenic GCN2 mutants), lots of cell-based read-outs not endogenous.

Comments on revised version.

The authors did a great job at addressing my initial critique on their manuscript and consequently I have no further comment.

# Referee report: Reviewer #3 (Public review)

**DOI:** 10.7554/eLife.108590.3.sa3

Summary:

In this manuscript, the authors describe the results of a high throughput screen for small molecule activators of GCN2. Ultimately, they find 3 promising compounds. One of these three, compound 20 (C20) is of the most interest both for its potency and specificity. The major new finding is that this molecule appears to activate GCN2 independent of GCN1, which suggests that it works by a potentially novel mechanism. Biochemical analysis suggests that each bind in the ATP binding pocket of GCN2, and that at least in vitro C20 is a potent agonist. Structural modeling provides insight into how the three compounds might dock in the pocket and generates testable hypotheses as to why C20 perhaps acts through a different mechanism than other molecules.

Strengths:

Of the 3 compounds identified by the authors, C20 is of the most interest, not just for its intriguing mechanistic distinction as being GCN1-independent (shown genetically in two distinct cell lines, CHO and 293T, and in contrast to other GCN2 activators) but also for its potency. Ultimately, C20 might be a tool for providing mechanistic insight into the details of GCN2 activation and regulation and could be exploited therapeutically.

Weaknesses:

The chief limitation of this work is that the experiments exploring the effects of C20 on ISR output in cells are limited, so how useful these compounds are both experimentally and therapeutically remains to be determined.

Comments on revised version.

The authors have satisfactorily addressed my comments. A more extensive analysis of UPR signaling in cells (transcription and cell death in particular) would have further strengthened the paper, but that can be left to future work.

# Author comment: Author response

**DOI:** 10.7554/eLife.108590.3.sa4

**Authors:** JiaYi Zhu, Giulia Emanuelli, Glenn Robert Masson, Vanessa Vinciauskaite, Henriette Willems, Peiqiao Cai, Andrew Lim, Christopher Alan Brown, David Winpenny, Murray Clarke, Rebecca Gilley, Fergus Preston, Jordan Wilson, Aldo Bader, Taufiq Rahman, Joseph E Chambers, John Skidmore, Nicholas Morrell, Stefan J Marciniak

**Affiliations:** University of Cambridge Cambridge United Kingdom; University of Cambridge Cambridge United Kingdom; University of Dundee Dundee United Kingdom; University of Dundee Dundee United Kingdom; University of Cambridge Cambridge United Kingdom; University of Cambridge Cambridge United Kingdom; University of Cambridge Cambridge United Kingdom; University of Cambridge Cambridge United Kingdom; University of Cambridge Cambridge United Kingdom; University of Cambridge Cambridge United Kingdom; Babraham Instititute Cambridge United Kingdom; Domainex Ltd Cambridge United Kingdom; University of Cambridge Cambridge United Kingdom; University of Cambridge Cambridge United Kingdom; University of Cambridge Cambridge United Kingdom; University of Cambridge Cambridge United Kingdom; University of Cambridge Cambridge United Kingdom; University of Cambridge Cambridge United Kingdom; University of Cambridge Cambridge United Kingdom

The following is the authors’ response to the original reviews

**Public Reviews:**

**Reviewer #1 (Public review):**

Summary:

This manuscript describes a chemical screen for activators of the eIF2 kinase GCN2 (EIF2AK4) in the integrated stress response (ISR). Recently, reported inhibitors of GCN2 and other protein kinases have been shown at certain concentrations to paradoxically activate GCN2. The study uses CHO cells and ISR reporter screens to identify a number of GCN2 activator compounds, including a potent "compound 20." These activators have implications for the development of new therapies for ISR-related diseases. For example, although not directly pursued in this study, these GCN2 activators could be helpful for the treatment of PVOD, which is reported for patients with certain GCN2 loss-of-function mutations. The identified activators are also suggested to engage with the GCN2 directly and can function while devoid of GCN1, a co-activator of GCN2.

Strengths:

The manuscript appears to be a largely rigorous study that flows in a logical manner. The topic is interesting and significant.

Weaknesses:

Portions of the manuscript are not fully clear. Some experimental presentation and design concerns should be addressed to support the stated conclusions.

We thank the reviewer for their supportive comments. We agree that portions of the manuscript were not fully clear and that some aspects of the experimental presentation and design required clarification.

To address this, we have revised the manuscript to make the experimental logic more transparent. In particular, we now explain more clearly the rationale for the screening strategy, including the use of histidinol as a canonical GCN2 activator, latrunculin A as a modulator of PPP1R15A-mediated eIF2α dephosphorylation, and tunicamycin as a PERK-dependent ER-stress control. We also clarify why a submaximal concentration of histidinol was used: this was intended to reveal compounds that enhance ISR signalling when GCN2 is partially activated.

We have clarified the use of the two ATF4 reporter systems. The ATF4–NanoLuc reporter was used for sensitive primary screening, whereas the ATF4–luc2 reporter was used as a more stringent orthogonal assay to prioritise robust ISR activators. We now state explicitly why some initial hits were not retained after testing in the second reporter line, and why the NanoLuc system was subsequently used again for mechanistic experiments.

Finally, we have revised the presentation of the orthogonal validation steps to make clearer how they support the stated conclusions. These include assays designed to distinguish GCN2-dependent ISR activation from indirect activation through ER stress, additional analysis of GCN2 dependence, and clearer interpretation of biochemical and docking data.

We hope that these revisions address the reviewer’s concern that the experimental design and data presentation needed to be made clearer in order to support the conclusions.

**Reviewer #2 (Public review):**

Summary:

In this manuscript, Zhu, Emanuelli, and colleagues describe a novel pharmacological activator of the Integrated Stress Response kinase GCN2. The work is conclusive and biochemically solid. This work significantly adds to the pharmacological arsenal targeting the ISR and, in particular, GCN2.

Strengths:

Strong biochemistry, novel molecular activator of GCN2 (GCN1 independent).

Weaknesses:

The rationale for the screen is not exploited in the results (e.g., pathogenic GCN2 mutants), and lots of cell-based read-outs are not endogenous.

We thank this reviewer for their positive assessment of the work. We address the three major concerns in turn below.

Major points

(1) Regarding the justification of the work. Since the authors justify the screen for GCN2 activators with loss-of-function mutants associated with diseases, it would be of interest to evaluate whether the best compounds identified in the study are indeed able to prompt activation of those mutants (or at least of the most prevalent). This approach could actually go in parallel with the docking experiments carried out in the last figure of the manuscript, where mutants could be modelized as well.

To address this point, we tested whether the lead compounds could activate disease-associated GCN2 variants linked to pulmonary veno-occlusive disease. In contrast to GCN2iB, the new compounds did not activate these variants. We now state this explicitly in the manuscript, thereby clarifying that although the compounds identify a new mode of GCN2 activation, they do not rescue the pathogenic GCN2 variants tested here.

Results

“Moreover, in contrast to GCN2iB (17), the current compounds did not activate disease-associated GCN2 variants linked to PVOD [data not shown].”

(2) The compounds are only tested using « artificial » proximal signaling outputs. It would be interesting to evaluate whether the best identified compounds are capable of prompting endogenous eIF2alpha phosphorylation in cellular models.

We thank the reviewer for this suggestion. Detecting eIF2α phosphorylation following activation of GCN2 is technically challenging and typically produces weaker signals compared to activation of other ISR kinases, such as PERK (e.g. by thapsigargin). For this reason, many studies rely on downstream reporter assays to monitor GCN2 activity. To address the reviewer’s concern, we have now included an orthogonal readout of ISR activation by assessing global translation using a puromycin incorporation assay. Using this approach, we show that compound 20 significantly reduces translation, and importantly, this effect is attenuated in GCN2-deficient cells, supporting a GCN2-dependent mechanism.

Results

“Studies with compound 18 were limited by poor aqueous solubility; therefore, time‑course analyses focused on compounds 20 and 21. To assess ISR activation over an extended period, live‑cell luciferase measurements were performed using CHO cells stably expressing an ATF4::Nanoluc-PEST reporter. Both compounds elicited maximal reporter activation between 6 and 8 h (Figure S1A&B). Compound 20, but not 21, induced a significant GCN2‑dependent reduction in mRNA translation, as measured by puromycin incorporation, with a progressive effect observed up to 7 h (Figure S1C-F).”

(3) Other GCN2 activators (other than GCN2iB, e.g., HC-7366) were recently identified. In this context, it would be of interest to carry out a small benchmarking study to evaluate how the compounds identified in the current study perform against the previously identified molecules.

We thank the reviewer for this suggestion. In response, we obtained HC-7366 and assessed its activity alongside our compounds in the CHO ATF4::NanoLuc reporter assay. In this system, compound 20 demonstrated greater potency than HC-7366 (see reviewer figure below). However, we note that HC-7366 showed relatively limited activity in CHO cells in our hands, despite previously reported strong effects in other cellular systems and in vivo models. This context-dependent activity makes direct benchmarking difficult. Accordingly, we have included this comparison in the revised manuscript and discuss this limitation in the Discussion.

Discussion

“We also evaluated the reported GCN2 activator HC-7366 in our CHO ATF4::NanoLuc reporter system. In this context, HC-7366 showed limited activity relative to compound 20, despite its reported efficacy in other cellular systems and in vivo (data not shown). This highlights potential context dependence in small‑molecule activation of GCN2 and limits direct cross-study comparison.”

**Author response image 1.** ISR activation by compound 20 and GC-7366 in CHO cells. Normalised fold-change in ATF4 signal in CHO ATF4::NanoLuc reporter cells treated for 19 hours with Compound 20 or HC-7366. DMSO was used as vehicle control. (representative experiment, mean ± SEM, n=3 technical replicates).

**Reviewer #3 (Public review):**

Summary:

In this manuscript, the authors describe the results of a high-throughput screen for small-molecule activators of GCN2. Ultimately, they find 3 promising compounds. One of these three, compound 20 (C20), is of the most interest both for its potency and specificity. The major new finding is that this molecule appears to activate GCN2 independent of GCN1, which suggests that it works by a potentially novel mechanism. Biochemical analysis suggests that each binds in the ATP-binding pocket of GCN2, and that at least in vitro, C20 is a potent agonist. Structural modeling provides insight into how the three compounds might dock in the pocket and generates testable hypotheses as to why C20 perhaps acts through a different mechanism than other molecules.

We agree that GCN1-independent activation suggests a potentially distinct mechanism of action. While we are currently unable to define the mechanistic basis underlying the GCN1-independence of compound 20, prior work provides some relevant context. Recent studies have shown that the ATP-competitive modulator GCN2iB can activate GCN2 independently of GCN1 under specific conditions, notably in the context of the GCN2 E26A mutant [Carlson, 2023]. This observation raises the possibility that, under certain conditions, engagement of the kinase domain, potentially via the ATP-binding pocket, may bypass the requirement for GCN1. However, in our system, we did not observe GCN1-independent activation with GCN2iB at the concentrations tested. This discrepancy may reflect a narrow or context-dependent window for such activity, or differences between wild-type and mutant GCN2. These findings suggest that GCN1-independent activation of GCN2 may occur under specific conditions or with distinct classes of compounds, although further work will be required to define the underlying mechanism for compound 20. We have added the following to the main text:

Discussion

“Recent work suggests that the ATP-competitive modulator GCN2iB can activate GCN2 independently of GCN1 under specific conditions using a GCN2 E26A mutant (9). In our hands, we did not observe GCN1‑independent activation with GCN2iB at the concentrations tested. This discrepancy may reflect a narrow concentration window for GCN1‑independent activation or context‑dependent effects of the E26A mutation. These findings raise the possibility that GCN1‑independent activation of GCN2 may occur under specific conditions or with distinct classes of compounds.”

Strengths:

Of the 3 compounds identified by the authors, C20 is the most interesting, not just for its intriguing mechanistic distinction as being GCN1-independent (shown genetically in two distinct cell lines, CHO and 293T in Figure 4, and in contrast to other GCN2 activators) but also for its potency. In in-cellulo assays, compound 21 appears as more of an ISR enhancer than an activator per se, and although compound 18 and compound 21 lead to upregulation of the ISR targets (Figure 2), that degree of upregulation is probably not significantly different from that induced by those compounds in Gcn2-/- cells. For C20, the effect appears stronger (although it is unclear whether the authors performed statistical analysis comparing the two genotypes in Figure 2D). In Figure 3, only C20 activates the ISR robustly in both CHO and 293T. Ultimately, C20 might be a tool for providing mechanistic insight into the details of GCN2 activation and regulation, and could be exploited therapeutically.

Prompted by this suggestion, we assessed C20 in two additional commonly used cell lines: human colon carcinoma HCT116 cells and African green monkey COS7 cells. C20 showed no activity in these models. In contrast, primary mesothelioma cells (Mesobank T12) exhibited robust PPP1R15A induction in response to the compound. The following text has been added to the manuscript.

Results

“We went on to examine downstream cellular consequences of GCN2 activation in multiple models. While compounds did not induce detectable ISR signalling in HCT116 or COS‑7 cells under the conditions tested, induction of PPP1R15A was observed in Mesobank T12 primary mesothelioma cells, indicating context-dependent biological responses… [data not shown].”

Weaknesses:

There are some limitations to the existing work. As the authors acknowledge, they do not use any of the compounds in animals; their in vivo efficacy, toxicity, and pharmacokinetics are unknown. But even in the context of the in cellulo experiments, it is puzzling that none of the three compounds, including C20, has any effects in HeLa cells when Neratinib does. It's beyond the scope of this paper to address definitively why that is, but it would at least be reassuring to know that C20 activates the ISR in a wider range of cells, including ideally some primary, non-immortalized cells. In addition, the ISR is a complex, feedback-regulated response whose output varies depending on the time point examined. The in cellulo analysis in this paper is limited to reporter assays at 18 hours and qRT-PCR assays at 4 and 8 hours. A more extensive examination of the behaviour of the relevant ISR mRNAs and proteins (eIF2, ATF4, CHOP, cell viability, etc.) for C20 across a more extensive time course would give the reader a clearer sense of how this molecule affects ISR output.

We thank the reviewer for this insightful suggestion. To address the need for a more comprehensive assessment of ISR signalling, we have extended our analysis across a broader time course and incorporated additional functional readouts. Using the ATF4-NanoLuc reporter, compounds 20 and 21 exhibit peak ISR activation at approximately 6-8 h in wild-type cells. In parallel, we assessed global mRNA translation using puromycin incorporation and found that compound 20, but not compound 21, induces a progressive reduction in translation over this period, which is dependent on GCN2. While we agree that direct measurement of upstream ISR markers such as eIF2α phosphorylation can be informative, detection of GCN2-mediated eIF2α phosphorylation is technically challenging and often less robust than activation of other ISR kinases (e.g. PERK). For this reason, we have prioritised orthogonal downstream functional readouts, including reporter activity and translational output, to capture ISR pathway engagement. These additional data provide a clearer picture of the kinetics and functional consequences of compound-induced ISR activation and have been incorporated into the revised manuscript.

Results

“Studies with compound 18 were limited by poor aqueous solubility; therefore, time‑course analyses focused on compounds 20 and 21. To assess ISR activation over an extended period, live‑cell luciferase measurements were performed using CHO cells stably expressing an ATF4::Nanoluc-PEST reporter. Both compounds elicited maximal reporter activation between 6 and 8 h (Figure S1A&B). Compound 20, but not 21, induced a significant GCN2‑dependent reduction in mRNA translation, as measured by puromycin incorporation, with a progressive effect observed up to 7 h (Figure S1C-F).”

I also find it a bit strange that the authors describe C20 as "demonstrat(ing) weak inhibition of ... PKR" - the measured IC50 is ~4 μM, which is right around its EC50 for GCN2 activation. This raises the confounding possibility that C20 would simultaneously activate GCN2 while inhibiting PKR. While perhaps inhibition of PKR is not relevant under the conditions when GCN2 would be activated either experimentally or therapeutically, examining in cells the effects of C20 on GCN2 and PKR across a dose range would shed light on whether this cross-reactivity is likely to be of concern.

We thank the reviewer for highlighting compound 20 as the most interesting lead compound and for recognising its apparent ability to activate GCN2 independently of GCN1. The reviewer identified several limitations relating to cell-type specificity, the temporal behaviour of ISR activation, and possible PKR cross-reactivity.

In response to the concern about cell-type specificity, we tested compound 20 in additional cellular models. Compound 20 did not induce detectable ISR signalling in HCT116 or COS-7 cells under the conditions tested, consistent with the reviewer’s observation that activity is not universal across cell types. However, we observed induction of PPP1R15A in primary Mesobank T12 mesothelioma cells. We have therefore revised the manuscript to present compound 20 activity as cell-context dependent rather than broadly generalisable across all cell types.

To address the reviewer’s concern that the ISR output was examined only at limited time points, we extended the time-course analysis for compounds 20 and 21. Using live-cell ATF4::NanoLuc reporter measurements, both compounds showed maximal reporter activation at approximately 6-8 hours. We also measured translational output by puromycin incorporation and found that compound 20, but not compound 21, caused a progressive GCN2-dependent reduction in translation. These data provide a clearer view of the kinetics and functional consequences of compound 20-mediated ISR activation.

The reviewer also noted that compound 20 inhibits PKR in vitro at concentrations close to those required for GCN2 activation in cells. We agree that this is an important potential liability. Because PKR signalling was not robustly or reproducibly inducible in our CHO-based reporter system, we were unable to perform a reliable cellular dose-response analysis of PKR engagement in the present study. We have therefore revised the Discussion to acknowledge kinase cross-reactivity, including possible PKR inhibition, as an important limitation and an issue for future development of this chemical series.

Finally, we have moderated our mechanistic interpretation of compound 20. Although the data support direct engagement of GCN2 and suggest a mechanism distinct from canonical GCN1-dependent activation, we now discuss GCN1-independent activation more cautiously and in the context of prior reports that GCN2iB can display GCN1-independent activity under specific experimental conditions.

Discussion

“While the functional relevance of PKR inhibition in our cellular systems is uncertain, these observations highlight the potential for kinase cross-reactivity, which will be important to address in future studies.”

**Recommendations for the authors:**

**Reviewer #1 (Recommendations for the authors):**

(1) The description of the chemical screen for Gcn2 activators is not sufficiently clear and detailed. (a) Briefly and early on provide the rationales (modes of action) for using histindinol and latruculin A. Explain further the rationale in Figure 2, outlining the purpose for the combined compound + submaximal dose of histindinol.

The text has been amended.

Results

“Histidinol activates GCN2 by inhibiting histidyl‑tRNA synthetase, leading to the accumulation of uncharged tRNAHis. This uncharged tRNA binds to GCN2, relieving its autoinhibition and activating the kinase (35). Latrunculin A sequesters G‑actin, thereby inhibiting PPP1R15A activity (36, 37). Tunicamycin inhibits protein glycosylation in the endoplasmic reticulum (ER), resulting in activation of PERK (38).”

“This submaximal concentration was used to allow detection of compounds that enhance ISR signalling when GCN2 is partially activated.”

b) What is unique about the second CHO: ATF4-luc2 reporter line? Why do only 89 out of the original 130 compounds induce the ISR in this line versus the original CHO: ATF4-Nanoluc cell line? This is confusing for the reader about how compounds were triaged for characterization.

The ATF4‑Nanoluc and ATF4‑luc2 reporter lines differ only in the luciferase used, but this has important practical consequences. The Nanoluc reporter is substantially more sensitive, so it was used for the primary screen to detect even weak ISR activation. The luc2 reporter has lower sensitivity and a narrower dynamic range, making it a more stringent orthogonal assay. As a result, not all hits from the Nanoluc screen (130 compounds) reproduced in the luc2 line; the 89 compounds retained are those that robustly activate the ISR under these more stringent conditions. This step was therefore used to prioritise stronger, more reproducible activators for downstream characterisation.

Results

“While primary screening was performed in ATF4‑Nanoluc lines for maximal sensitivity, hits were subsequently re-tested in a second CHO ATF4::luc2 reporter line as a more stringent orthogonal assay to prioritise robust ISR activators. Of the 130 hits identified in the sensitive Nanoluc screen and passing early toxicity assessment, 89 were confirmed in the luc2 assay, consistent with enrichment for higher-amplitude ISR activators under more stringent detection conditions.”

c) The study uses a second CHO reporter line in the flow scheme (CHO:ATF4-luc2) and then switches back to an ATF4-Nanoluc line to establish GCN2 dependence. What is the rationale for switching back to the original reporter line?

The luc2 reporter line was used as a more stringent, orthogonal validation step to prioritise robust ISR activators. For subsequent mechanistic studies, including assessment of GCN2 dependence, we returned to the ATF4‑Nanoluc line because its higher sensitivity and simpler single‑reagent assay format are better suited to multi‑point measurements and comparative analyses. In effect, the luc2 reporter was used for triage, whereas the Nanoluc system was retained for mechanistic characterisation and downstream screening.

Results

“In subsequent mechanistic studies, the ATF4‑Nanoluc reporter was again used to take advantage of its higher sensitivity and simpler assay format for multi‑condition comparisons.”

d) The rationale for the first orthogonal screen described in the results section to identify inducers of ER stress is not clearly explained. The compounds were already determined to be dependent on GCN2 prior to this test, and one would have thought that this criterion would have covered ER stress and alternative eIF2 kinase activators.

We agree with the reviewer that, in principle, establishing GCN2 dependence should reduce the likelihood of capturing compounds acting through alternative eIF2α kinases. However, we performed this orthogonal ER stress screen to address two practical considerations. First, high‑throughput screening is inherently prone to false positives, as it is typically conducted at a single concentration and time point, and compound libraries may contain degraded or chemically inconsistent material. We therefore used a lower‑throughput, more controlled ER stress assay with freshly sourced compounds and additional readouts (e.g. CHOP and XBP1) to improve confidence in the hits. Second, despite prior evidence of GCN2 dependence, ER stress signalling via PERK converges on the same downstream endpoints: eIF2α phosphorylation and ATF4 induction. We therefore wished to explicitly exclude compounds that activate the ISR indirectly via ER stress. In practice, this proved important, as the orthogonal assay did identify compounds that induced ER stress, which we subsequently excluded from the lead set.

Results

“Although hits were prioritised for GCN2 dependence, we performed an additional orthogonal screen to exclude compounds that activate the ISR indirectly via ER stress, which converges on the same downstream outputs.”

(2) A major point of the manuscript is that there is GCN1 independence for the small molecule activation of GCN2, and this has not yet been reported. One report for this GCN1 independence is reference 9 [Carlson … Wek 2023] (Figure 5). In this report, low doses of GCN2iB that can activate GCN2 (although by the present manuscript at much lower levels than the identified new compounds) induce ATF4 expression in cells expressing an E26A mutant of GCN2 that is suggested to negate GCN1 binding and enhancement of GCN2 activity. Halofuginone induction of ATF4 expression was thwarted by the GCN2 E26A mutant.

We thank the reviewer for highlighting this important point. We agree that Carlson et al. (2023) suggest that, under certain conditions, GCN2iB can activate GCN2 independently of GCN1 using the E26A mutant. In our experiments, however, we did not observe GCN1‑independent activation with GCN2iB under the conditions tested, i.e. similar low doses. One possible explanation is that the GCN1‑independent activity reported by Carlson et al. occurs only within a narrow concentration range; their observations were made at very low compound concentrations, whereas higher concentrations may engage additional regulatory mechanisms. In our study, we used concentrations optimised for robust ISR activation, which may mask such effects. We also note that the E26A mutation (E18A in yeast), originally identified by two‑hybrid analysis, disrupts the GCN2-GCN1 interaction but may not completely eliminate all modes of functional coupling under all conditions. Taken together, these observations raise the possibility that GCN1‑independent activation represents a context‑dependent mechanism that may be unmasked only under specific experimental conditions or by particular classes of compounds.

We have revised the Discussion to acknowledge this prior report explicitly and to clarify how our findings relate to it.

Discussion

“Recent work suggests that the ATP-competitive modulator GCN2iB can activate GCN2 independently of GCN1 under specific conditions using a GCN2 E26A mutant (9). In our hands, we did not observe GCN1‑independent activation with GCN2iB at the concentrations tested. This discrepancy may reflect a narrow concentration window for GCN1‑independent activation or context‑dependent effects of the E26A mutation. These findings raise the possibility that GCN1‑independent activation of GCN2 may occur under specific conditions or with distinct classes of compounds.”

(3) The authors state that the ISR was exaggerated in Ppp1r15a KO cells. It would be helpful to include statistical analyses to support this statement.

Thank you for enabling us to be more precise. New text added:

Results

“Activation of the ISR by tunicamycin was exaggerated in the *Ppp1r15a^-/-^* cells owing to their defective dephosphorylation of eIF2a (wild type vs *Ppp1r15a^-/-^*, p<0.05).”

(4) The results state that Chop and Ppp1r15a mRNAs were measured following 4 hours of treatment with compound 18, 20, or 21, but Figure 2D shows treatment from 0 to 8 hours? It appears that the compound still induces these mRNAs in GCN2 KO cells, possibly with delayed kinetics. A lengthened time course study would help determine if this is indeed the case.

We thank the reviewer for this careful observation. To address the reviewer’s point regarding delayed or GCN2‑independent signalling, we have extended our analysis using compounds 20 and 21, which are more tractable experimentally (solubility). Using the ATF4‑Nanoluc reporter, both compounds show peak ISR activation at ~6–8 h in wild-type cells over an extended time course. In parallel, functional readouts of mRNA translation (puromycin incorporation) demonstrate that compound 20, but not 21, induces a progressive, GCN2‑dependent reduction in translation over this period. These clarify the temporal aspects of signalling by these two compounds.

Results

“Studies with compound 18 were limited by poor aqueous solubility; therefore, time‑course analyses focused on compounds 20 and 21. To assess ISR activation over an extended period, live‑cell luciferase measurements were performed using CHO cells stably expressing an ATF4::Nanoluc-PEST reporter. Both compounds elicited maximal reporter activation between 6 and 8 h (Figure S1A&B). Compound 20, but not 21, induced a significant GCN2‑dependent reduction in mRNA translation, as measured by puromycin incorporation, with a progressive effect observed up to 7 h (Figure S1C-F).”

Legend

“Supplementary Figure S1. Kinetics of responses to compounds 20 and 21

(A-B) Wild-type CHO cells stably expressing the ATF4::nanoLuc-PEST reporter were treated with Nano-Glo and either (A) 13mM compound 20 or (B) 13mM compound 21. Median bioluminescence (fold change normalised to DMSO control) ± 95% confidence. Representative experiment (n=4 technical repeats). (C-F) Representative immunoblot of lysates from wild-type or *Eif2ak4^-/-^* CHO cells treated with 10μM compound 20 or 7.5μM 21 for the indicated times. Immediately before harvesting, cells were treated with 10μg/mL puromycin to label newly synthesised polypeptides. “-“ indicates cells not incubated with puromycin. “U” cells were treated with puromycin but without test compound. “CHX” represents the cycloheximide control (100μg/mL). Molecular size in kDa. (E-F) Quantification of puromycinylated proteins normalised to GAPDH. Mean ± SEM. CHO WT (black) and *Eif2ak4^-/-^* cells turquoise. N = 4 independent experiments. Two-way ANOVA with Šídák's multiple comparisons test; ***: p ≤ 0.001.”

(5) In the section describing the differences between cell lines in the ability of compounds to induce the ISR, this is difficult for the reader to interpret, as no controls are included. How does histidinol (or other canonical inducers of the ISR) behave in the three reporter assays (CHO, 293T, and HeLa)?

As requested, we now provide ATF4::Nanoluc reporter activation (3mM, 20 hours because of this drug’s slow kinetics)

Results

“To benchmark ISR activation in these models, each cell type was treated with 3mM histidinol (Figure S2). Reporter activation was most robust in CHO cells, followed by 293T cells, then HeLa cells.”

Discussion

“Moreover, histidinol-induced ISR activation showed a clear hierarchy across cell lines, with CHO cells being the most responsive and HeLa cells the least.”

Legend

“Supplementary Figure S2. Cell-type differences in response to histidinol

Fold-change of ATF4::NanoLuc reporter signal in HEK293T, HeLa and CHO cells transiently transfected with reporter and treated for 20 hours with 3mM histidinol. Fold-change calculated relative to vehicle control. Mean ± SEM.”

We thank the reviewer for this important question. However, we respectfully disagree that a direct correspondence between the concentrations required for target engagement in the BRET assay and for ISR activation in functional assays should necessarily be expected. BRET (including NanoBRET) is a target engagement assay that measures compound binding to the protein in intact cells, typically by competition with a labelled tracer, and thus reports on apparent intracellular affinity and occupancy rather than downstream biological effect (Robers 2019, PMID 30519940). By contrast, ISR activation is a functional readout that reflects amplification through signalling networks, and can be influenced by multiple additional variables including pathway non-linearity, feedback, and kinase regulation. Consequently, it is well established that potencies derived from target engagement assays do not always align with those measured in functional assays. For example, intracellular kinase profiling studies using NanoBRET have demonstrated systematic potency offsets between binding/engagement measurements and downstream cellular activity, arising from factors such as intracellular ATP competition and pathway context (PMID Capener 2026, PMID 41495225). More generally, target engagement assays provide a quantitative measure of binding, whereas functional assays measure biological outcome, and these readouts need not coincide because they capture distinct aspects of a compound’s mechanism of action. Accordingly, we interpret our BRET data as evidence of direct interaction with GCN2 in cells, rather than as a predictor of the concentration required to activate the ISR. The observation that higher concentrations are required in the BRET assay is therefore not unexpected and does not argue against a requirement for kinase-domain engagement in ISR activation. Instead, it reflects the different mechanistic endpoints captured by the two assay formats.

We will clarify this point explicitly in the revised manuscript.

Results

“The concentrations required to detect target engagement in NanoBRET assays did not directly mirror those required for ISR activation, reflecting the distinction between ligand binding and downstream pathway output.”

(7) In Figure 5E, the authors suggest that compounds 18 and 20 are non-competitive inhibitors of GCN2 since the Vmax increases with increasing ATP concentration. What is the K^m^ for ATP in the absence or presence of compound 18 or 20? It would be helpful to include progress curves as supplementary data to support the Vmax plots in Fig. 5E. Consider providing more specific units (currently arbitrary units) for the y-axis.

We thank the reviewer for this insightful comment and agree that our original wording overstated the mechanistic interpretation of these data. In particular, the use of the term “non‑competitive” is not well supported by the current analysis and may be misleading, especially given that our data are consistent with binding within or proximal to the ATP-binding pocket. We have therefore revised the text to remove this designation and instead describe the data more conservatively in terms of changes in apparent Vmax, without assigning a specific inhibition mechanism. With respect to kinetic analysis, we agree that full determination of K^m^ values and inclusion of progress curves would provide a more rigorous mechanistic interpretation. However, given the primary focus of this manuscript on identifying and characterising small‑molecule activators of GCN2 in cells, we believe that a detailed steady‑state kinetic analysis would be beyond the scope of the current study. We have therefore moderated our conclusions accordingly and now present these data as preliminary kinetic observations rather than definitive evidence of inhibition modality.

Results

“Compounds 18 and 20 altered the apparent kinetic parameters of GCN2, including an increase in the observed V_max_; however, these data do not allow assignment of a specific inhibition modality.”

(8) The full-length GCN2 assay presented in Figure 5G appears to be unresponsive to uncharged tRNA, a known regulator of GCN2. The statement that compound 20 induces eIF2 phosphorylation to a greater extent than tRNAs is true for the in vitro assay, but arguably is because the in vitro assays do not recapitulate the in vivo arrangement.

We thank the reviewer for this comment. We respectfully disagree that the assay is unresponsive to uncharged tRNA. In our hands, GCN2 does exhibit activation in response to tRNA; however, the magnitude of this effect is modest (~4‑fold) compared to the substantially stronger activation observed with compound 20 (~40‑fold). As a result, the tRNA response can appear compressed when both are plotted on the same scale. We agree with the reviewer that the in vitro assay does not fully recapitulate the in vivo regulatory environment, where factors such as GCN1 and ribosome association are known to potentiate GCN2 activation. This limitation likely explains the relatively weaker response to tRNA under our assay conditions and was a key motivation for incorporating cellular assays in our study. Interestingly, the marked difference in activation magnitude between tRNA and compound 20 in vitro raises the possibility that compound-mediated activation may, at least in part, bypass regulatory features that normally constrain GCN2 activity in a GCN1‑dependent manner. While we have not directly tested this hypothesis, we will temper the wording and include this as a speculative point in the Discussion.

Results

“Of note, uncharged tRNA produced a modest (~4‑fold) activation of GCN2 under these conditions, whereas compound 20 induced substantially greater (~40‑fold) activation.”

Discussion

“The markedly greater activation observed with compound 20 compared with uncharged tRNA in vitro raises the possibility that such compounds may partially bypass regulatory constraints on GCN2 activation, including those normally mediated by GCN1.”

(9) Using purified GCN2 kinase domain at low ATP concentrations (10 μM), compounds 18 and 20 were shown not to inhibit GCN2 up to concentrations of 3 μM. In previous assays, much higher concentrations of compounds 18 and 20 were used to inhibit GCN2. Why were different concentrations used? This makes this interpretation of this data difficult for the reader to draw conclusions.

We thank the reviewer for this comment and agree that the use of different concentration ranges across assays may not have been sufficiently clear. The kinase‑domain assay performed at low ATP (10 μM) was specifically designed to assess whether compounds 18 and 20 have a propensity to inhibit GCN2 under conditions that sensitise detection of ATP‑competitive effects and facilitate comparison with related eIF2α kinases. This assay was therefore optimised for detecting inhibition, rather than activation. In contrast, the higher concentrations used in other experiments were selected to robustly measure ISR activation in cellular or full‑length protein contexts, where higher compound exposure is required to observe downstream signalling outputs. These two assay systems therefore address distinct mechanistic questions—targeting inhibition under controlled biochemical conditions versus activation in more complex functional settings—and are not directly comparable in terms of concentration–response relationships. We will revise the manuscript to clarify this distinction and to emphasise that the kinase‑domain assay was not intended to define the activation potency of the compounds.

Results

“This assay was performed at low ATP concentrations to sensitise detection of ATP-competitive inhibition and was not optimised to detect compound-mediated activation of GCN2.”

(10) In silico docking studies support the binding of compound 20 in the ATP-binding pocket of the GCN2 kinase domain. How is this compatible with the stated ATP non-competitive mechanism?

We agree with the reviewer that our previous description was misleading. The designation of compounds 18 and 20 as “ATP non‑competitive” is not supported by the available data and is inconsistent with the docking results suggesting binding within the ATP‑binding pocket. We have therefore revised the manuscript to remove this terminology and to describe the kinetic behaviour more cautiously, without assigning a specific mode of inhibition.

(11) The study does not appear to feature biological assays demonstrating the effects of GCN2 activation. For example, does compound 20 reduce translation or growth of cells in a GCN1/GCN2-dependent manner, or do the compounds overcome PVOD mutations akin to the authors' Hum Mol Genet 2024 Aug 18;33(17):1495-1505 article?

We thank the reviewer for this suggestion. We agree that defining downstream biological consequences of GCN2 activation is an important goal. We did explore this using several cellular systems; however, these effects were context-dependent and not consistently observed across models. Specifically, compounds did not induce a detectable ISR in HCT116 or COS‑7 cells under the conditions tested, despite responsiveness of these systems to canonical activators such as histidinol. In contrast, we did observe induction of PPP1R15A in primary mesothelioma cells, indicating that biological responses can be elicited in certain cellular contexts. We also tested whether these compounds could rescue disease-associated GCN2 variants linked to PVOD, as previously reported for GCN2iB, but did not observe activation of these mutants. These findings suggest that while the compounds robustly activate GCN2 signalling in reporter assays, downstream biological outputs are context-dependent and may require specific cellular conditions or co-factors. Given the variability across systems, we have limited our conclusions to ISR activation and have not generalised broader biological effects. We will clarify this point in the revised manuscript.

Results

“We went on to examine downstream cellular consequences of GCN2 activation in multiple models. While compounds did not induce detectable ISR signalling in HCT116 or COS‑7 cells under the conditions tested, induction of PPP1R15A was observed in Mesobank T12 primary mesothelioma cells, indicating context-dependent biological responses. Moreover, in contrast to GCN2iB (17), the current compounds did not activate disease-associated GCN2 variants linked to PVOD [data not shown].”

(12) For the control of neratinib and other activators linked with ATP binding that are suggested to be dependent on GCN1 in Fig. 4, include reporter induction by drug treatment in Gcn2-/- cells. It would be helpful to be clear in the list of compounds between those suggested to be direct activators versus those that may create stress that leads to GCN2 activation.

We thank the reviewer for this suggestion. In response, we have performed additional reporter assays in both WT and *GCN2^-^*/- cells to assess the dependence of drug-induced ISR activation on GCN2. These experiments reveal clear GCN2 dependence for reporter induction upon treatment with sunitinib, NXP800, WEE1-in-4, Debio0123, gefitinib, and erlotinib, as signal is markedly reduced in GCN2⁻/⁻ cells compared to WT.

In contrast, dovitinib and AZD1775 show less clear dependence, with relatively low reporter signal even in WT cells (notably lower than observed in Fig. 4C), limiting interpretation. Interestingly, dabrafenib induces stronger reporter activity in GCN2^-/-^ cells than in WT, indicating that its effects are independent of GCN2 and may reflect activation of alternative stress or signalling pathways.

Results

“To further assess the mechanism of compound-induced ISR activation, we evaluated reporter responses in GCN2-deleted cells (Supplementary Figure S3). Several compounds, including sunitinib, NXP800, WEE1-in-4, Debio0123, gefitinib, and erlotinib, showed reduced reporter activity in GCN2-deficient cells, consistent with GCN2-dependent activation. In contrast, dovitinib, AZD1775 and dabrafenib produced weaker or inconclusive responses even in the paired wild-type lines, limiting analysis. These data allow us to distinguish compounds consistent with direct or GCN2-dependent activation from those more likely to induce ISR indirectly through cellular stress upstream of GCN2. These findings support a distinction between compounds that activate the ISR through GCN2-dependent mechanisms and those that likely act indirectly via alternative stress pathways.”

Legend

“Supplementary Figure S3. GCN2-dependence of ISR activation by putative GCN2 agonists

Normalised fold-change in ATF4 signal in CHO WT (purple) and Gcn2-/- (blue) ATF4::NanoLuc reporter cells treated for 19 hours with a panel of ATP-competitive kinase inhibitors reported to activate GCN2 (at 1 and 3µM, sunitinib used at 3 and 10µM, AZD1175 used at 0.3 and 1µM, gefitinib and erlotinib used at 3 and 10µM). DMSO was used as vehicle control. (n=3; mean ± SEM).”

Minor Comments:

(1) In the introduction, the authors state that "Several type 1 and 1.5 kinase inhibitors can activate GCN2 at low concentrations while inhibiting at higher concentrations". What is the evidence that type I inhibitors can activate GCN2?

Thank you for this query. We believe our initial phrasing was open to misinterpretation. The new wording is

Introduction

“Several kinase inhibitors classified as type 1 or type 1.5 with respect to their canonical targets can activate GCN2 at low concentrations while inhibiting it at higher concentrations; however, their binding mode to GCN2 remains undefined.”

(2) A CHO:ATF4-Nanoluc translation reporter screen was used to screen 123K compounds and divide them into a pool of inhibitors and a pool of activators. The criteria used to make this distinction are not sufficiently described in the manuscript, and screen results are not supplied as supplementary data.

We thank the reviewer for highlighting the need for greater clarity regarding the screening criteria and reproducibility. Compounds from the primary screen (BioAscent library of 123,222 drug-like compounds performed by the ALBORADA Drug Discovery Institute) were classified based on Z-score thresholds, with activators defined as those with Z-score > 3. To assess robustness, the primary screen data were re-analysed independently. In an initial analysis of 121,000 compounds (excluding plates failing quality control), 6,521 compounds met the activator threshold. Of these, 6,461 overlapped with the original hit list. The small number of discrepancies included: (i) compounds absent from the analysed dataset (e.g. originating from failed plates), and (ii) compounds with Z-scores close to the threshold (typically between −3 and −3.02), consistent with minor analytical variation (e.g. rounding). Overall, these analyses show a high degree of concordance in hit identification, with differences restricted to borderline cases near the selection threshold. We have clarified these criteria below.

Results

“Compounds were classified based on Z-score thresholds derived from the primary screen, with activators defined as those with Z-score > 3. An initial analysis of 121,000 compounds, excluding plates failing quality control, identified 6,521 activators, of which 6,461 overlapped with the subset selected for follow-up screening. Minor discrepancies were restricted to compounds absent from the analysed dataset (e.g. originating from failed plates) or those with Z-scores close to the threshold (3 to 3.02), consistent with limited analytical variation. Using this approach, we assembled a subset of 6,461 compounds enriched for potential ISR activators and screened these in 384-well format at 10 µM for 16 hours.”

**Reviewer #3 (Recommendations for the authors):**

(1) The legend to Figure 4 should read "Compound 20 displays GCN1 independence", not "dependence".

Thank you for spotting this error. We have made the correction.

(2) The text describes Figure 2D as examining 4h, but it also examines 8h.

We have amended the text to:

“After validating their effects using the assays described above, we next confirmed activation of the ISR by these compounds at the transcriptional level at 4 and 8 hours.”

(3) Unless I'm misreading Table S1, compound Z134826202 is listed as activating the ISR, but the authors describe it in the text as inactive.

Thank you. We have corrected this error.
